# Identifying strategies to support implementation of interprofessional primary care teams in Nova Scotia: Results of a survey and knowledge sharing event

**DOI:** 10.1186/s12875-024-02399-0

**Published:** 2024-05-10

**Authors:** Amy Grant, Rachel Giacomantonio, Kelly Lackie, Adrian MacKenzie, Elizabeth Jeffers, Julia Kontak, Emily Gard Marshall, Susan Philpott, Debbie Sheppard-LeMoine, Elizabeth Lappin, Alice Bruce, Amy Mireault, Deanna Beck, Lindsay Cormier, Ruth Martin-Misener

**Affiliations:** 1 Maritime SPOR SUPPORT Unit, Nova Scotia Health, Nova Scotia, Canada; 2 School of Nursing, Dalhousie University, Nova Scotia, Canada; 3 Research, Innovation and Discovery, Nova Scotia Health, Nova Scotia, Canada; 4 WHO/PAHO Collaborating Centre On Health Workforce Planning & Research, Dalhousie University, Nova Scotia, Canada; 5 Present Address: Department of Health and Wellness, Government of Nova Scotia, Nova Scotia, Canada; 6 Department of Family Medicine, Dalhousie University, Nova Scotia, Canada; 7 Department of Nursing, St. Francis Xavier University, Nova Scotia, Canada; 8 Faculty of Nursing, University of Windsor, Ontario, Canada; 9 Primary Health Care and Chronic Disease Management Network, Nova Scotia Health, Nova Scotia, Canada; 10 School of Nursing, Faculty of Health, Dalhousie University, PO Box 15000, 5869 University Avenue, Halifax, NS B3H 4R2 Canada

**Keywords:** Collaborative family practice, Interprofessional teams, Primary care, Consolidated Framework for Implementation Research, Implementation, Workforce

## Abstract

**Background:**

Interprofessional primary care teams (IPCTs) work together to enhance care. Despite evidence on the benefits of IPCTs, implementation remains challenging. This research aims to 1) identify and prioritize barriers and enablers, and 2) co-develop team-level strategies to support IPCT implementation in Nova Scotia, Canada.

**Methods:**

Healthcare providers and staff of IPCTs were invited to complete an online survey to identify barriers and enablers, and the degree to which each item impacted the functioning of their team. Top ranked items were identified using the sum of frequency x impact for each response. A virtual knowledge sharing event was held to identify strategies to address local barriers and enablers that impact team functioning.

**Results:**

IPCT members (*n* = 117), with a mix of clinic roles and experience, completed the survey. The top three enablers identified were access to technological tools to support their role, standardized processes for using the technological tools, and having a team manager to coordinate collaboration. The top three barriers were limited opportunity for daily team communication, lack of conflict resolution strategies, and lack of capacity building opportunities. IPCT members, administrators, and patients attended the knowledge sharing event (*n* = 33). Five strategies were identified including: 1) balancing patient needs and provider scope of practice, 2) holding regular and accessible meetings, 3) supporting team development opportunities, 4) supporting professional development, and 5) supporting involvement in non-clinical activities.

**Interpretation:**

This research contextualized evidence to further understand local perspectives and experiences of barriers and enablers to the implementation of IPCTs. The knowledge exchange event identified actionable strategies that IPCTs and healthcare administrators can tailor to support teams and care for patients.

**Supplementary Information:**

The online version contains supplementary material available at 10.1186/s12875-024-02399-0.

## Introduction

Access to primary care (i.e., a regular primary care provider, timely access to care) in North America has been increasingly difficult over the past 20 years, with documented shortages in the primary care physician workforce [1–3]. In Canada, although the number of primary care physicians per citizen has increased over time [4], the amount of clinical activity has decreased [5, 6]. Concurrently, there has been an increase in patient demand given a growing population and increasing complexity of patient care needs [7–9]. The primary care system in Nova Scotia, Canada faces similar challenges [10, 11]. The number of people in the province who identify as needing a regular family practice provider has doubled over a 3-year period [12], with increases in all four geographic health service management zones, despite the provincial primary care workforce growing by 58 family physicians and 118 NPs during that time [13].

Improving access to primary care through the development of interprofessional teams has been a national goal since the early 2000s [14], with advocates recently calling for an expansion of team-based primary care for a system in crisis [15, 16]. Interprofessional Primary Care Teams (IPCTs) are an approach to the delivery of primary care that involves three or more healthcare providers (HCPs), at least two of whom are different professions (e.g. family physicians, nurse practitioners, social workers), who work interdependently to provide high-quality patient care [17]. IPCTs reduce wait times, improve care coordination, contribute to more appropriate referrals, reduce duplication of services and emergency department visits [18–20], improve patient outcomes, and reduce HCP burnout [21–24]. In Nova Scotia, IPCTs have demonstrated positive impacts on accessibility [25, 26], chronic disease prevention and management [27], and patient satisfaction [26].

Despite challenges in accessing primary care [12, 28] and calls for increasing the number of and support for IPCTs, implementation has varied across Canada [29, 30] and internationally [30–32], both in how quickly teams have been implemented [33–35] and the mix of HCPs included [36]. Implementation strategies that are responsive to local contexts [37], or tailored to individual, team, or policy levels [38–41], have greater uptake [41].

Our team conducted a literature review to identify theoretically-informed barriers and enablers to IPCT implementation [42], using the Consolidated Framework for Implementation Research (CFIR) [43]. Most barriers and enablers were categorized into two domains of the CFIR, *II. Outer Setting* (which referred to Government, Health Authorities and Health Organizations in the context of our research), and *III. Inner Setting* (which referred to Characteristics of the Team in our research). Key themes identified within the *Outer* Setting were around professional renumeration plans, regulatory policy and interprofessional education. Within the *Inner Setting*, key themes focused on team-leadership (e.g., having a manager responsible for day-to-day activities), clear governance, technology that supports information sharing amongst the team, and clear and consistent communication. Building on this completed literature review, the current study aimed to support the continued implementation of IPCTs by 1) identifying and prioritizing barriers to and enablers of implementation by IPCT team members, and 2) co-creating team-level strategies to mitigate and/or enhance the prioritized barriers and enablers, respectively, through a knowledge sharing event.

## Methods

This study was performed in accordance with the Declaration of Helsinki and approved by the appropriate ethics committee. Ethics approval was obtained from Nova Scotia Health (NSH), Research Ethics Board (Approval #1,026,183). For the survey portion of the study, consent was implied by opening and completing the survey, which was described in the information provided to potential participants. For the knowledge sharing event, the need for informed consent was waived by the ethics board as the nature of the event involved mutual sharing of information and co-development of implementation strategies. All methods were carried out in accordance with relevant guidelines and regulations.

### Aim I: Survey to identify and prioritize barriers and enablers

#### Survey development

**Fig. 1:**
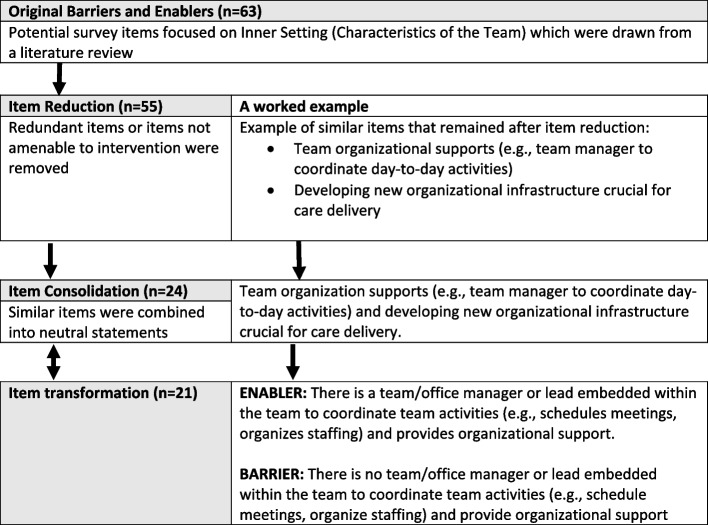
Survey item reduction and development process with a worked example

Barriers and enablers to IPCT implementation were identified via a literature review [42] using the CFIR [43], which the research team used to create the survey (Appendix A). Survey items were identified through a three-step process of item reduction, consolidation, and transformation (Fig. 1). The survey focused on items within *Domain III – Inner setting* or *Characteristics of the Team* to detect strategies that could be enacted at the practice level.

The barriers and enablers identified in the literature review were combined into shared concepts and consolidated into opposing barrier and enabler statements (*n* = 21) to prompt respondents to identify whether they had experienced each item as a barrier, enabler, or neither. In the second stage of the survey, respondents rated selected items on a 5-point Likert scale (1 = no impact to 5 = significant impact). The survey also contained an open-ended question on barriers and enablers to IPCT implementation. Demographic information (e.g., role, time with team) was also collected.

#### Survey recruitment

An online survey was administered using Research Electronic Data Capture (REDCap), a secure web-based electronic data capture tool designed for research studies, hosted at Nova Scotia Health [44, 45]. Members of IPCTs (*n* = 85 teams at the time of the survey) in Nova Scotia including HCPs, managers, administrative staff, and health service leads (for role definitions see: https://cfpt.nshealth.ca/team-members) were invited to participate. Although the exact number of staff working on teams is not available, the most recent data available estimates there were 377 family physicians working on collaborative family practice teams close to the time of the survey [46], with teams having a minimum of 3 people with at least two having different roles [47], and most having fewer than 10 staff [48]. The maximum possible sample size was estimated to be between 600 to 850 people.

Health care in Nova Scotia is planned provincially but implemented locally. At Nova Scotia Health, there are four geographic health service management zones. To facilitate broad recruitment across the province, the Director of Primary Health Care in each health service management zone sent emails to Health Service Managers and Health Service Leads who then invited ICPT teams they manage to complete the survey. Three reminder emails were sent at two-week intervals [49]. Targeted recruitment was used when there was a low response rate within a zone or from specific professions to maximize the number of respondents. This involved managers and/or leads sending a more directive follow-up email to highlight the low response rate from the zone or from specific health professions (e.g., administrators) to encourage participation. Respondents were also offered a chance to win one of five $100 gift cards.

#### Survey data analysis

Data were analyzed using SPSSV26.0 [50]. Demographic information and questionnaire responses were summarized using descriptive statistics. For each potential barrier and facilitator, a sum score was generated from the product of its frequency (number of respondents who indicated they had experienced the item) and its impact (response item selected on the 5-point Likert scale). The summed scores for each statement were compared across participant roles and other demographics, and combined scores were used to determine prioritization rankings. Responses to the open-ended question were analyzed deductively to the CFIR domains by one team member (SA) and inductively using content analysis to identify overarching themes [51]. Results are reported in accordance with the Checklist for Reporting Results of Internet E-Surveys (CHERRIES) [52].

### Aim II: Co-creating strategies through a knowledge sharing event

A two-hour, virtual knowledge sharing event was held on October 20, 2022 to: 1) share survey findings and 2) co-create strategies to mitigate and/or enhance priority barriers and enablers.

#### Recruitment for knowledge sharing event

Recruitment was purposive to attract participation from IPCT HCPs and staff, Primary Health Care Leads and Managers, patients and caregivers, and government representatives. Invitations were emailed by Zone Directors to Zone Health Service Managers to IPCTs. Existing Patient and Family Advisors and MSSU Patient Public Partners were also emailed invitations by Patient Engagement Advisors. Participants completed an online registration that collected information about their roles and where they work to help assign individuals to breakout groups. Prior to the event, participants were sent the event objectives, agenda, and discussion topics.

#### Event structure

Following an overview of the literature review and survey results, participants were split into pre-assigned groups, with a mix of participants based on role and practice location, for world café-style discussions [53]. Experienced interprofessional facilitators were each assigned one topic: team organization and coordination supports; communication tools and technology; role clarity and relationships; goals and feedback; or availability of resources and leadership engagement. Each topic was associated with priority barriers and enablers, and a set of prompt questions (Appendix B). Each facilitator met with two breakout groups, such that each breakout group had the opportunity to discuss two topics. Following the event, participants were invited to complete an online event evaluation survey using Select Survey v5.0 [54]. Participants responded to statements about the event objectives and possible applications on 5-point Likert scale ranging from strongly agree to strongly disagree or very likely to very unlikely. Responses were collapsed into agree (i.e., strongly agree, agree), neutral, or disagree (i.e., disagree, strongly disagree).

#### Knowledge sharing event analysis

A content analysis of audio/video recordings of breakout group discussions identified overarching themes, strategies, and actions to address the barriers and enablers discussed [51]. Five team members independently coded breakout group discussions for one topic (AG, AB, AMir, RG, EL), and met to compare their analyses, and to revise and agree on the coding. Two team members (AB, AMir) independently coded the next recording, and then again met to compare results and discuss with the coding team. The remaining topics were double-coded (AB, AMir). Discrepancies were resolved by group consensus. Findings were consolidated into strategies and actions by one team member (AB) and were reviewed by the full study team.

## Results

### Aim I: Survey to identify and prioritize barriers and enablers

The survey was partially (*n* = 94) or fully (*n* = 93) completed by 187 respondents. Respondents who only completed the demographic portion of the survey were excluded from the analysis (*n* = 70). Although we do not have data to determine proportional representation of the survey respondents to the total number of ICPT staff, or to the number of roles across ICPTs, we estimate that those who responded to the survey (and whose data was included) represent between 23 to 29% of total ICPT members in the province, matching our expected response rate of 30%. Respondents’ demographic characteristics are summarized in Table 1. The top three enablers and barriers are identified in Table 2. The top three enablers were related to technological tools and organizational supports and the top three barriers were communication and information sharing, team culture and climate, and education and training.

**Table 1:** Demographic characteristics of survey respondents

	**Respondents** (*N* = 117)
	N (%)
**Respondent profession**	
RN/FPN/LPN	26 (22.2)
NP	16 (13.7)
GP	34 (29.1)
Admin Assistant	21 (17.9)
Clinic Manager	4 (3.4)
Social Worker	5 (4.3)
Dietitian	4 (3.4)
Other	7 (6.0)
**# years in Practice**	
< 1	4 (3.4)
1–5	37 (31.6)
6–10	22 (18.8)
11–15	21 (17.9)
16–20	10 (8.5)
> 20	22 (18.8)
**# years on IPCT**	
< 1	12 (10.3)
1–5	82 (70.1)
6–10	9 (7.7)
11–15	9 (7.7)
16–20	2 (1.7)
**# years IPCT in existence**	
< 1	3 (2.6)
1–5	61 (52.1)
6–10	23 (19.7)
11–15	16 (13.7)
16–20	7 (6.0)
> 20	5 (4.3)
**Other roles on team reported by respondents**	
RN	66 (56.4)
FPN	56 (47.9)
LPN	20 (17.1)
NP	82 (70.1)
GP	99 (84.6)
Admin. Assistant	88 (75.2)
Clinic Manager	64 (54.7)
Social Worker	41 (35.0)
Psychologist	4 (3.4)
Physiotherapist	7 (6.0)
Occupational therapist	2 (1.7)
Dietitian	39 (33.3)
Other +	26 (22.2)
**Governance Model**	
Unsure	44 (37.6)
Contracted Services	24 (20.5)
Co-leadership	38 (32.5)
Turn-key	10 (8.5)
**Zone**	
Central	57 (48.7)
Western	20 (17.1)
Eastern	24 (20.5)
Northern	15 (12.8)

^*^NP Nurse Practitioner, *FPN* Family Practice Nurse, *GP* General/Family Physician; #Other included Health Services Lead/Manager, Pharmacist, Podiatrist. *% is expressed out of the total sample size, as not all respondents completed demographic questions; + Other included psychiatrist, urologist, pharmacist, podiatrist, specialist, addiction

**Table 2.**
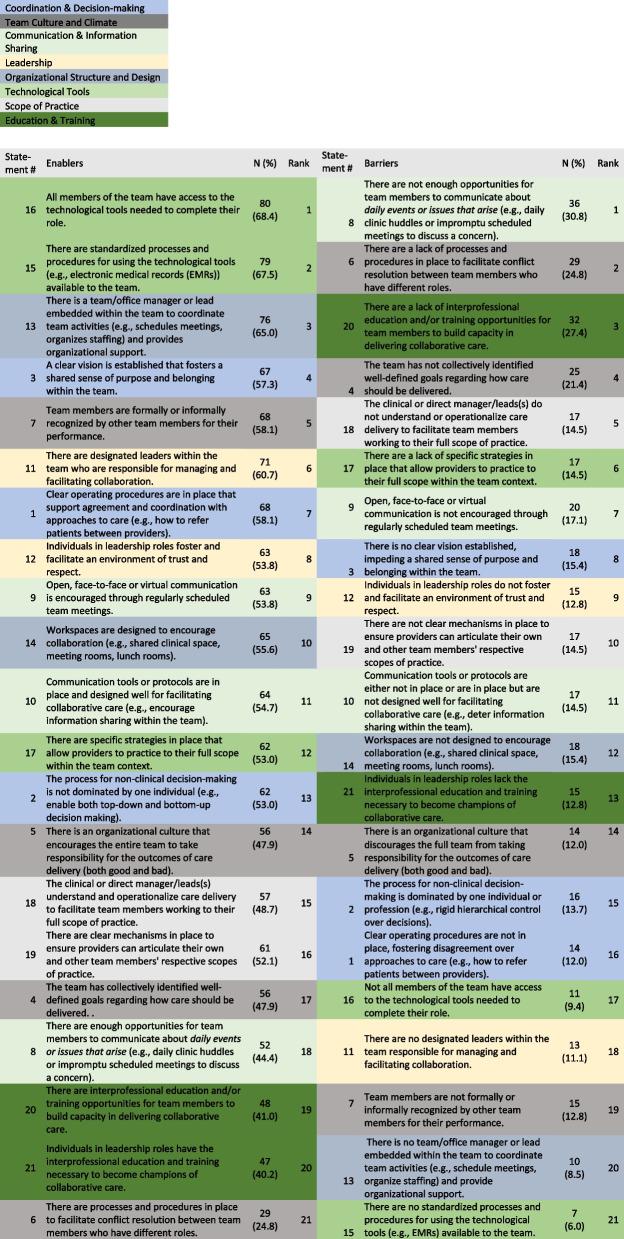
Barriers and enablers with associated rank

The top ranked barriers and enablers were compared across participant roles (Table 3). The ranking of the top three enablers was similar across participant roles, however there were differences in rankings across the remaining items. For example, nurses and administrators/managers identified the importance of clear operating procedures (Statement 1) in their top 7, whereas this was ranked 13 by medical doctors (MDs). There were similar rankings across the top three barriers. MDs ranked items related to collaborative care and scope of practice (Statements 10, 19) higher than other respondents. Conversely, administrators/managers ranked items about leadership and organizational supports (Statements 12, 13) more highly than those in clinical roles.

**Table 3:** Comparison of ranking across participant roles

	**Enablers (rank)**				**Barriers (rank)**			
**Statement #**	**Nurses**	**GPs**	**Admin/** **Manager**	**Other**	**Nurses**	**GPs**	**Admin/** **Manager**	**Other**
1	4	13	6	9	15	16	14	9
2	9	18	16	6	20	17	7	16
3	7	10	4	8	14	9	3	11
4	17	17	9	11	4	3	4	6
5	20	12	15	16	16	8	11	14
6	21	21	20	21	3	1	1	5
7	5	7	5	12	18	19	10	12
8	19	5	18	20	1	2	2	1
9	8	8	14	15	10	11	12	4
10	10	14	8	10	12	5	13	8
11	6	9	11	2	17	12	16	15
12	11	6	19	1	9	13	5	19
13	3	3	10	4	21	14	6	7
14	18	4	2	17	6	20	21	10
15	1	2	3	7	19	21	18	18
16	2	1	1	3	11	15	17	20
17	14	11	12	5	7	10	15	13
18	12	16	13	13	5	18	8	17
19	13	15	7	19	13	4	19	3
20	15	19	21	18	2	6	9	2
21	16	20	17	14	8	7	20	21

Twenty-three respondents (20%) answered an open-ended question about barriers and enablers that were not part of the pre-defined survey statements. Themes identified included: leadership (importance of trust and respect), funding models (fee-for-service models impacting time for collaboration), and the built environment (shared space) (Table 4).

**Table 4:** Qualitative analysis of open-ended survey responses

Theme	Description	Sample quote
Leadership (*n* = 4)	Differences in levels of competency and involvement by co-leaders can be a barrier to collaboration	*In the co-leadership model, there is a clinical lead and an organizational lead. Some of the enablers are grounded by a strong clinical lead despite having poor organizational leadership. Several of the barriers are impacted by poor organizational leadership that is not outweighed by good clinical leadership. For example, barriers around scope of practice are primarily influenced by organizational leadership while enablers about fostering trust and respect are driven almost exclusively by clinical leadership.* (Nurse)
Funding model (*n* = 3)	The Fee for Service (FFS) funding model was identified as a barrier to collaborative practice as it creates a disincentive for physicians to collaborate as they lose revenue	*Barriers include the fee for service model within a collaborative practice. Physicians are 'scared' to give up their patient care as they won’t be able to bill for some visits.* (Nurse)
Built Environment (*n* = 2)	The workspace was both a facilitator and a barrier to collaborative practice	*Enabler: shared team lounge/lunchroom—allows for informal collaboration and team building.* (GP) *Having a bigger working area would be beneficial as we run out of space often. Organization around office is key and run is limited*. (Admin)

The top 10 barriers and enablers from the survey were grouped into five categories and discussed at the knowledge sharing event (Appendix B).

### Aim II: Co-creating strategies through a knowledge sharing event

Thirty-three stakeholders participated in the knowledge sharing event, with a mix of roles and health service management zones represented (Table 5).

**Table 5:** Participants’ demographics

	**Participants (*N* = 33)** **n (%)**
**Role**	
Family Physician	4 (12)
Nurse Practitioner	4 (12)
Registered Nurse and/or Family Practice Nurse	2 (6)
Clinic Manager / Administrators	2 (6)
Health Service Managers/Leads	6 (18)
Patient and/or caregiver attached to a CFPT	5 (15)
Other	10 (30)
**Nova Scotia Health Management Zones**	
Central	7 (21)
Northern	7 (21)
Eastern	0 (0)
Western	19 (58)

Four overarching themes were identified: 1) Considering and consulting the community to address community and patient needs alongside the needs of the practice; 2) Tailoring implementation strategies and approaches to the needs of individual clinics; 3) Clear and consistent communication is crucial and requires dedicated resources; and 4) Practice governance and funding models need to be designed to support team collaboration. Each of these themes represent considerations that support multiple implementation strategies and impact all levels of implementation (patients and caregivers; individual providers; teams; and policy and organizations). Five multi-modal implementation strategies with 26 associated actions were identified during breakout group discussions (Table 6). A visual summary of these themes, strategies, and actions is available online.

**Table 6:** Summary of implementation strategies and associated actions

Implementation Strategy	Description	Associated Actions
Optimize scope of practice to balance patient care and provider needs	This strategy supports team members to work flexibly within their scope of practice, balancing the needs and interests of the provider when assigning patient services. This approach was seen as favourable to always working to full scope of practice, which could concentrate challenging cases with physicians and nurse practitioners, and contribute to staff burnout. Balancing patients appointments amongst various staff roles was also seen to build trust between patients and the whole team, prior to the onset of serious health concerns, and helps familiarize patients on the role of different clinic staff and how their functions overlap	• Allow providers to be flexible in working to full scope of practice (balance provider workload and reduce burnout) • Build positive rapport and trust between the patients and the whole team • Include Patient and Family Advisors (PFAs) and patients as stakeholders to the practice • Provide education within the clinic and to the public about team members and their respective roles, abilities, and scopes of practice • Balance the abilities and interests of team members so patients can be scheduled to an appropriate provider (may increase access to care) • Incorporate technology and software that makes patient files accessible to all team members (may facilitate care and case conference)
Holding regular and accessible meetings	This strategy highlights the importance of using meetings as a medium of communication and support for all staff within the practice. Different formats and frequencies can be used strategically to support practice goals and activities	• Be respectful of members’ time during meetings (have an agenda, meeting goal(s), keep to time) • Use meetings to communicate practice needs and share feedback, and discuss barriers experienced by team members and the community • Include all members of the practice in team meetings for transparency, to facilitate collaboration, understand patient needs, and provider scope (e.g., administrative staff) • Choose a consistent virtual communication software for ease of use (e.g., Zoom, Skype, Teams) • Use meeting strategically to support various practice goals and activities (e.g., roundtables, meetings with other community providers, patient case-conferences) • Establish protected time for team meetings
Support team development opportunities	This strategy focuses on facilitating and improving teamwork within the practice	• Model collaboration behaviour for other team members • Support team members in working together rather than independently • Ensure team members know that they’re appreciated (e.g., rewarding good work) and share success stories to boost morale • Educate team members on governance models and how they affect teamwork (e.g., union requirements, different contractual obligations) • Discuss collaborative strategies experiences by team members in other settings (e.g., in school) and how they can be included in the practice • Allot recurring time to discuss practice goals, quality standards, and revisit the memorandum of agreement • Provide a medium for anonymous feedback by team members and patients • Create a leadership role responsible for collaboration and effective teamwork • Design physical spaces to facilitate and encourage teamwork
Support professional development opportunities	This strategy focuses on additional training for individual team members and how it can benefit the practice as well as practitioners	• Encourage and support mentorship within the practice, allowing members to share skills and grow their scope of practice • Provide and support opportunities for team members to build skills through educational opportunities • Consider practice composition when hiring new staff (e.g., mentoring opportunities)
Support involvement in non-clinical activities	This strategy captures the challenges with billing and compensation experienced by team members who use a "fee for service" model, which makes it difficult for members to bill for professional time not spent on direct patient care	• Use the funding available for collaborative activities and, when possible, have administrative staff complete the Family Physician Collaboration Payment Form • Create payment mechanisms that compensate all team members for collaborative activities including attending regular meetings, without the need for additional billing requests

The post-event survey was fully or partially completed by 18 event participants (54.5%) (Table 7). Most respondents (83%) agreed that they gained a greater understanding of the barriers and enablers to IPCT implementation and heard perspectives they otherwise would not have heard (82%). Similary, most respondents felt that they engaged with others to brainstorm strategies (76%) and that the event provided an effective means of doing so (71%). However, of those who responded to questions about application, fewer respondents indicated that they were likely to apply strategies identified through the event (69%).

**Table 7:** Evaluation survey responses

	**Survey Respondents** **n (%)**
**Role (*n* = 18)**	
Family Physician	2 (11)
Nurse Practitioner	4 (22)
Registered Nurse and/or Family Practice Nurse	0
Clinic Manager / Administrators	2 (11)
Health Service Managers/Leads	2 (11)
Patient and/or caregiver attached to a CFPT	4 (22)
Other	4 (22)
**Opportunities for dialogue**	
**Have a greater understanding of barriers/enablers to implementation (*n* = 18)**	
Agree	15 (83)
Neutral	2 (11)
Disagree	1 (6)
**Heard perspectives they may not have otherwise heard (*n* = 17)**	
Agree	14 (82)
Neutral	3 (18)
Disagree	0 (0)
**Strategy co-creation**	
**Engaged with others to brainstorm potential strategies (*n* = 17)**	
Agree	13(76)
Neutral	4(24)
Disagree	0(0)
**Event was an effective way to support brainstorming strategies (*n* = 17)**	
Agree	12(71)
Neutral	5(29)
Disagree	0(0)
**Application**	
**How likely are you apply any of the recommendations identified through this event (*n* = 13)**	
Likely	9 (69)
Neutral	4 (31)
Unlikely	0 (0)
**Do you feel the strategies identified have the potential to improve patient care (*n* = 3)**	
Likely	3 (75)
Neutral	0 (0)
Unlikely	0 (0)

## Interpretation

This research prioritized barriers and enablers, and co-developed team-level strategies to support implementation of IPCTs in Nova Scotia. To our knowledge, this is the first research to collect contextually relevant data on barriers and enablers to IPCTs in the province. An estimated 23 to 29% of ICPT staff responded to the survey, with adequate representation from many of the main roles on teams (e.g., physicians, nurses, administrative support), and broad representation across the province. Top enablers identified by IPCT members were related to technological tools (e.g., EMRs) and management supports (e.g., having a team or office manager to coordinate team activities, leaders who can manage and facilitate collaboration). Top barriers focused on communication, including limited opportunities to discuss daily events or issues that arise (e.g., lack of daily clinic huddles or scheduled meetings), and a lack of processes and procedures to resolve conflicts specifically between team members who have different roles. A lack of interprofessional training opportunities was also identified as a top barrier. There were some differences in the ranking of barriers and enablers across professional roles, likely due to the perspective each role brings to the team and the daily challenges they face based on the nature of their role. For example, nurses, physicians, and administrative support all ranked standardized processes and procedures for using technological tools in their top 3 enablers, whereas ‘other’ roles primarily composed of health service managers ranked this in their top 10. In another example, administrative staff ranked the importance of a team office manager lower on their list compared to other roles – perhaps not realizing how valued their own role is on the team. Despite some unique differences in how individuals perceived the importance of different barriers and enablers, there was a good degree of consistency in the top ranked items across roles, reflecting congruity amongst the team members.

The survey findings reflect the broader literature around barriers and enablers identified using the CFIR in our recent narrative review [42]. This review identified many barriers and enablers related to *Networks & Communication* including things like communication processes and tools (e.g., interprofessional care plans, common patient charts), access to electronic medical records, which were ranked as top barriers and enablers in the survey. The survey findings identified technological tools as a top enabler, while communication was a top barrier. *Available resources* were also frequently identified in the literature, which was reflected in the survey findings but focused specifically on the enabling function of management supports within the team. Open-ended data from the survey identified a few themes that added further contextual information around local experience. Some of this information, though informative, reflected on elements of the CFIR that were beyond the scope of this study (i.e., not on features of the team) – specifically referring to fee for service funding models and the built environment. These were extensively identified in the literature review but were not included as barriers or enablers in the current study given that they were not as easily amenable to a research-led intervention. Thus, our survey findings provide information on what barriers and enablers are relevant locally and will enable more focused intervention and supports.

The knowledge sharing event provided a low-cost, casual forum [55] for local primary care stakeholders to co-create actionable strategies that IPCTs and healthcare administrators can tailor to support teams and care for patients. The five strategies and 26 associated actions identified focus on optimizing scopes of practice to balance patient care needs and HCPs ability to meet those needs, having regular and accessible interprofessional meetings, supporting team and professional development, as well as finding ways to support the work involved in non-clinical administrative activities. No priority was assigned to strategies or related actions given that there is need to tailor strategies during implementation [56, 57]. Rather, these strategies serve as options for team members and stakeholders (e.g., health service managers) to consider for their particular practice conditions. The need to further tailor actions to practice needs may also explain why fewer respondents indicated an intention to apply strategies on the event evaluation—not all strategies will be appropriate for all settings and, as indicated by some participants, some strategies have already been implemented within IPCTs. The evaluation survey may therefore be biased towards respondents from practices that are already functioning quite well or who felt the strategies discussed in their breakout groups were directly relevant to their practices.

Despite the focus on team-based factors, several actions were associated with patients and caregivers. These actions clustered primarily within a single strategy, ‘Optimize scope of practice to balance patient care and provider needs,’ and focused on gathering patient and caregiver perspectives and providing a medium for anonymous feedback. Discussions about actions involving patients highlighted the importance of building trust and different but complimentary motivations for recommending specific strategies. For example, when discussing a desire to avoid physicians always working to their full scope of practice, patients voiced the importance of developing relationships with physicians prior to having a serious health concern, while clinicians and health service managers cited the need to avoid burnout. This reflects patients’ openness to being treated by various practice members [20], but also provides an example of how patient perspectives can help to optimize scope of practice and enable patient-centred care [24, 58]. Future research could aim to identify how best to incorporate patient and caregiver perspectives into the implementation of IPCTs.

The role of leadership in creating a culture of collaboration to support change was also identified as an enabler in the survey yet was not discussed at the knowledge sharing event. Since the discussion topics focused on team-level functions, this may have directed conversation away from individual actions and leadership. This gap may also be partially attributable to recruitment bias, as participants tended to describe positive experiences with well-functioning teams.

## Limitations

This review focused on features of the team, however, change is needed in other domains of the CFIR such as the outer setting (i.e., policy/health authority), or at the individual level where more personalized interventions would need to be developed. The strategies and actions identified provide a useful starting point for IPCTs to determine which strategies are most appropriate in their setting, when or how often to implement a change [55], and to refine the action during implementation [59]. Study recruitment was a challenge, as it was difficult to find an appropriate time during the COVID-19 pandemic to both launch a survey and to host a collaborative event, as primary care and health care workers were under pressure. This likely contributed to the incompletion rate of the survey, as providers may have been curious as to what the survey entailed but may have not had time to complete the remainder of the survey. Despite this, our response rate (23 to 29%) was in line with what we expected (30%) and we were able to recruit a mix of professional roles common in NS IPCTs at the time, with varying practice characteristics and fairly broad geographic representation. However, there are other professional roles not represented at the knowledge sharing event, for example social workers, dieticians, pharmacists, or medical learners. These roles are less common and absent on the majority of ICPTs in the province. Although we do expect that much of this research will still be applicable to a broader group of professionals, it is possible that some strategies may not reflect the experiences of these healthcare providers and warrant additional research in the future. Additionally, there may have been bias in who participated in the study, as the relationship between participants and managers and health service leads who sent the invitations may have influenced decisions about participation, or disclosure of criticism about team functioning. The response rate to the post-event evaluation survey was quite high (over 50%) however, the sample size is quite low and may not be representative of all ICPT members or roles. It is also possible that those who participated represented well-functioning teams whose positions afforded them the time to participate in these non-clinical activities.

## Conclusions

There is currently a strong focus on improving implementation of IPCTs both nationally [60] and provincially, which focuses on accessing care from the right provider, at the right time [61]. Given increasing issues with primary care access, with 15% of the provincial population currently waiting for a primary care provider [62], the need to focus on evidence-informed ways to improve implementation of IPCTs has never been more timely. The top enablers identified locally were reflective of the broader literature and included the importance of access to technological tools to share information on patients between team members and having strong management in place to facilitate team collaboration. Barriers were focused on lack of daily communication and conflict resolution. Through the knowledge sharing event, participants identified strategies to mitigate barriers and enhance enablers. These included 1) balancing patient needs and provider scope of practice, 2) holding regular and accessible meetings, 3) supporting team development opportunities, 4) supporting professional development, and 5) supporting involvement in non-clinical activities. These findings provide interprofessional, theoretically informed evidence about priority barriers and enablers of IPCT implementation in Nova Scotia, as well as a set of co-developed implementation strategies and actions that can be tailored to enhance implementation.

### Supplementary Information

**Supplementary Material 1**.

## Data Availability

All data generated or analyzed during this study are included in this published article [and its supplementary information files].
