# Supplementary material for: Identifying strategies to support implementation of interprofessional primary care teams in Nova Scotia: Results of a survey and knowledge sharing event
Source: BMC Prim Care. 2024 May 10;25:162. doi: 10.1186/s12875-024-02399-0 (PMC11083844; doi:10.1186/s12875-024-02399-0)
Supplement: Supplementary file 1 — Supplementary Material 1. [file 12875_2024_2399_MOESM1_ESM.docx]

**Appendix A: Barrier and Enabler Survey Questions**

21 statements will be presented in total. For each statement, please indicate whether you have experienced this concept as an enabler, a barrier, or neither to the functioning of *your current IPCT*.

| **Statement Category** | **Yes – I**  **have experienced it as an enabler (it has helped/improved)** | **Yes – I**  **have experienced it as a barrier (it has challenged/ prevented)** | **NEITHER:** I have not experienced this as either a barrier or an enabler (i.e., it has had no impact). |
| --- | --- | --- | --- |
| **Coordination & Decision-making** | 1a) **ENABLER:** Clear operating procedures **are in place that support agreement and coordination** with approaches to care (e.g., how to refer patients between providers). | 1b) **BARRIER:** Clear operating procedures **are not in place, fostering** **disagreement** over approaches to care (e.g., how to refer patients between providers). |  |
|  | 2a) **ENABLER:** The process for non-clinical decision-making is **not dominated by one individual** (e.g., enable both top-down and bottom-up decision making). | 2b) **BARRIER:** The process for non-clinical decision-making is **dominated by one individual or profession** (e.g., rigid hierarchical control over decisions). |  |
| **Team Culture and Climate** | 3a) **ENABLER:** A clear **vision is established** that **fosters** a shared sense of purpose and belonging within the team. | 3b) **BARRIER:** There is **no** clear vision established, **impeding** a shared sense of purpose and belonging within the team. |  |
|  | 4a) **ENABLER:** The team **has** collectively identified well-defined goals regarding how care should be delivered. | 4b) **BARRIER:** The team **has not** collectively identified well-defined goals regarding how care should be delivered. |  |
|  | 5a) **ENABLER:** There is an organizational culture that **encourages** the entire team to take responsibility for the outcomes of care delivery (both good and bad). | 5b) **BARRIER:** There is an organizational culture that **discourages** the full team from taking responsibility for the outcomes of care delivery (both good and bad). |  |
|  | 6a) **ENABLER:** There **are** processes and procedures in place to facilitate conflict resolution between team members who have different roles. | 6b) **BARRIER:** There are **a lack of** processes and procedures in place to facilitate conflict resolution between team members who have different roles. |  |
|  | 7a) **ENABLER:** Team members **are** formally or informally recognized by other team members for their performance. | 7b) **BARRIER:** Team members **are not** formally or informally recognized by other team members for their performance. |  |
| **Communication& Information Sharing** | 8a) **ENABLER:** There **are enough** opportunities for team members to communicate about *daily events or issues that arise* (e.g., daily clinic huddles or impromptu scheduled meetings to discuss a concern). | 8b) **BARRIER:** There **are not enough** opportunities for team members to communicate about *daily events or issues that arise* (e.g., daily clinic huddles or impromptu scheduled meetings to discuss a concern). |  |
|  | 9a) **ENABLER:** Open, face-to-face or virtual communication **is encouraged** through regularly scheduled team meetings. | 9b) **BARRIER:** Open, face-to-face or virtual communication **is not encouraged** through regularly scheduled team meetings. |  |
|  | 10a) **ENABLER:** Communication tools or protocols **are in place and designed well** for facilitating collaborative care (e.g., encourage information sharing within the team). | 10b) **BARRIER:** Communication tools or protocols **are either not in place** or **are in place but are not designed well** for facilitating collaborative care (e.g., deter information sharing within the team). |  |
| **Leadership** | 11a) **ENABLER:** There **are** designated leaders within the team who are responsible for managing and facilitating collaboration. | 11b) **BARRIER:** There **are no** designated leaders within the team responsible for managing and facilitating collaboration. |  |
|  | 12a) **ENABLER: Individuals in** leadership roles **foster and facilitate** an environment of trust and respect. | 12b) **BARRIER: Individuals in** leadership roles **do not foster and facilitate** an environment of trust and respect. |  |
| **Organizational Structure and Design** | 13a) **ENABLER:** There **is a** team/office manager or lead embedded within the team to coordinate team activities (e.g., schedules meetings, organizes staffing) and provides organizational support. | 13b)  **BARRIER: There is no** team/office manager or lead embedded within the team to coordinate team activities (e.g., schedule meetings, organize staffing) and provide organizational support. |  |
|  | 14a) **ENABLER:** Workspaces **are** designed to encourage collaboration (e.g., shared clinical space, meeting rooms, lunch rooms). | 14b) **BARRIER:** Workspaces **are not** designed to encourage collaboration (e.g., shared clinical space, meeting rooms, lunch rooms). |  |
| **Technological Tools** | 15a) **ENABLER:** There **are** standardized processes and procedures for using the technological tools (e.g., EMRs) available to the team. | 15b) **BARRIER:** There **are no** standardized processes and procedures for using the technological tools (e.g., EMRs) available to the team. |  |
|  | 16a) **ENABLER:** All members of the team **have access** to the technological tools needed to complete their role. | 16b) **BARRIER: Not** all members of the team **have** access to the technological tools needed to complete their role. |  |
| **Scope of Practice** | 17a) **ENABLER:** There **are** specific strategies in place that allow providers to practice to their full scope within the team context. | 17b) **BARRIER:** There **are a lack of** specific strategies in place that allow providers to practice to their full scope within the team context. |  |
|  | 18a) **ENABLER:** The clinical or direct manager/leads(s) **understand and operationalize** care delivery to facilitate team members working to their full scope of practice. | 18b) **BARRIER:** The clinical or direct manager/leads(s) **do not** **understand or operationalize** care delivery to facilitate team members working to their full scope of practice. |  |
|  | 19a) **ENABLER:** There **are** clear mechanisms in place to ensure providers can articulate their own and other team members' respective scopes of practice. | 19b) **BARRIER:** There **are not** clear mechanisms in place to ensure providers can articulate their own and other team members' respective scopes of practice. |  |
| **Education & Training** | 20a) **ENABLER:** There **are** interprofessional education and/or training opportunities for team members to build capacity in delivering collaborative care. | 20b) **BARRIER:** There **are** **a lack of** interprofessional education and/or training opportunities for team members to build capacity in delivering collaborative care. |  |
|  | 21a) **ENABLER:** Individuals in leadership roles **have** the interprofessional education and training necessary to become champions of collaborative care. | 21b) **BARRIER:** Individuals in leadership roles **lack** the interprofessional education and training necessary to become champions of collaborative care. |  |

The following list includes statements that you have experienced as a barrier or enabler from the previous question. For each statement, please indicate how it has impacted the functioning of your IPCT by rating it from 0 (indicating no impact), to 5 (indicating significant impact).

| **STATEMENTS** |  |  |  |  |  |
| --- | --- | --- | --- | --- | --- |
|  | No impact | Low impact | Moderate impact | High impact | Significant impact |
|  | 1 | 2 | 3 | 4 | 5 |
| *Statements selected above will be populated in this matrix question* |  |  |  |  |  |
|  |  |  |  |  |  |

4. Please provide any additional information you would like to share about the enablers and/or barriers that you have experienced, including any that were not in the statements provided. Suggestions or recommendations for enhancing the enablers or addressing the barriers are also welcome.

Insert your answer here

**Appendix B: Priority topics for the knowledge sharing event**

| **Topic** | | **Enablers** | **Barriers** | **Prompt Questions** |
| --- | --- | --- | --- | --- |
| **T1** | **Team Organization and Coordination Supports** | There is a team/office manager or lead embedded within the team to coordinate team activities (e.g., schedules meetings, organizes staffing) and provides organizational support. | There are a lack of specific strategies in place that allow providers to practice to their full scope within the team context. | - What strategies would support enablers to help team members work to their full scope of practice? - How could team members collaborate to support different professions to work to their full scope of practice? - How can the role of managers or leaders be improved or enhanced to support the team? |
| **T2** | **Communication Tools and Technology** | Open, face-to-face or virtual communication is encouraged through regularly scheduled team meetings.    All members of the team have access to the technological tools needed to complete their role.  There are standardized processes and procedures for using the technological tools (e.g., EMRs) available to the team. | Open, face-to-face or virtual communication is not encouraged through regularly scheduled team meetings.    There are not enough opportunities for team members to communicate about daily events or issues that arise (e.g., daily clinic huddles or impromptu scheduled meetings to discuss a concern). | - What are some strategies or supports needed to help teams communicate regularly? - What structures and processes could be created to support daily communication among team members? - How can technology be used to continue to enhance the work of IPCTs? - Are there new ways current technology can be used support the need for open communication? |
| **T3** | **Role Clarity and Relationships** | Clear operating procedures are in place that support agreement and coordination with approaches to care (e.g., how to refer patients between providers). | There are a lack of processes and procedures in place to facilitate conflict resolution between team members who have different roles.    The clinical or direct manager/leads(s) do not understand or operationalize care delivery to facilitate team members working to their full scope of practice.    There are not clear mechanisms in place to ensure providers can articulate their own and other team members' respective scopes of practice. | - What strategies could team leaders put in place to support team members to work to their full scope of practice? - What strategies are required to help team leaders and health care providers learn about each professions scope of practice? - What education, processes or procedures should be put in place to support interprofessional conflict resolution between team members? - How can operating procedures that support team members to collaborate be improved or supported to continue? |
| **T4** | **Goals and Feedback** | A clear vision is established that fosters a shared sense of purpose and belonging within the team.    Team members are formally or informally recognized by other team members for their performance. | The team has not collectively identified well-defined goals regarding how care should be delivered.    There is no clear vision established, impeding a shared sense of purpose and belonging within the team. | - How can teams be supported to develop of a clear vision for their work? - If a team has a clear vision for their work, how can this be supported to continue? - How can the team work together to develop goals for how care should be delivered? What processes could be put in place to support this? - How can team members be recognized for their performance? How can this be made a sustainable part of team practice? |
| **T5** | **Readiness for Implementation (Availability of Resources and Leadership Engagement)** | Workspaces are designed to encourage collaboration (e.g., shared clinical space, meeting rooms, lunch rooms).    There are designated leaders within the team who are responsible for managing and facilitating collaboration. | There are a lack of interprofessional education and/or training opportunities for team members to build capacity in delivering collaborative care. | - What interprofessional education or training opportunities would support team members to build capacity in collaborative care? - How can leaders be supported to continue to manage and facilitate team collaboration? - What strategies would support leaders to engage in interprofessional education? - How can workspaces be enhanced or changed to encourage collaboration? |
